# Metabolomic profiling reveals a role for CPT1c in neuronal oxidative metabolism

**DOI:** 10.1186/1471-2091-13-23

**Published:** 2012-10-25

**Authors:** Jieun Lee, Michael J Wolfgang

**Affiliations:** 1 Department of Biological Chemistry, Center for Metabolism and Obesity Research, Johns Hopkins University School of Medicine, Baltimore, MD, 21205, USA

## Abstract

**Background:**

Carnitine Palmitoyltransferase-1c (CPT1c) is a neuron specific homologue of the carnitine acyltransferase family of enzymes. CPT1 isoenzymes transfer long chain acyl groups to carnitine. This constitutes a rate setting step for mitochondrial fatty acid beta-oxidation by facilitating the initial step in acyl transfer to the mitochondrial matrix. In general, neurons do not heavily utilize fatty acids for bioenergetic needs and definitive enzymatic activity has been unable to be demonstrated for CPT1c. Although there are studies suggesting an enzymatic role of CPT1c, its role in neurochemistry remains elusive.

**Results:**

In order to better understand how CPT1c functions in neural metabolism, we performed unbiased metabolomic profiling on wild-type (WT) and CPT1c knockout (KO) mouse brains. Consistent with the notion that CPT1c is not involved in fatty acid beta-oxidation, there were no changes in metabolites associated with fatty acid oxidation. Endocannabinoids were suppressed in the CPT1c KO, which may explain the suppression of food intake seen in CPT1c KO mice. Although products of beta-oxidation were unchanged, small changes in carnitine and carnitine metabolites were observed. Finally, we observed changes in redox homeostasis including a greater than 2-fold increase in oxidized glutathione. This indicates that CPT1c may play a role in neural oxidative metabolism.

**Conclusions:**

Steady-state metabolomic analysis of CPT1c WT and KO mouse brains identified a small number of metabolites that differed between CPT1c WT and KO mice. The subtle changes in a broad range of metabolites *in vivo* indicate that CPT1c does not play a significant or required role in fatty acid oxidation; however, it could play an alternative role in neuronal oxidative metabolism.

## Background

Although the mammalian brain is lipid rich and mutations in lipid metabolizing enzymes result in debilitating neurological disease, neurons are generally not thought to rely on mitochondrial fatty acid beta-oxidation for bioenergetic requirements. Neurons instead mainly utilize the oxidation of glucose for most of their bioenergetic needs, although, during prolonged fasting, ketone bodies (i.e. acetoacetate and beta hydroxybutyrate) can also be used [1]. Most neurons have a low amount of the rate-setting enzymes in mitochondrial long chain fatty acid catabolism, namely, the malonyl-CoA sensitive Carnitine Palmitoyltransferase 1 (CPT1a and CPT1b) enzymes which limit most neurons potential for mitochondrial fatty acid beta-oxidation [2].

Carnitine acyltransferases are enzymes that catalyze the exchange of acyl groups between carnitine and Coenzyme A (CoA) to facilitate the transport acyl chains between the cytoplasm to the mitochondrial matrix [3]. CPT1 isoenzymes (EC 2.3.1.21) preferentially are positioned on the outer mitochondrial membrane and transfer long chain acyl groups from CoA to carnitine. CPT1a and CPT1b are malonyl-CoA sensitive and therefore inhibited when malonyl-CoA levels are high (e.g. during high glucose flux). The malonyl-CoA insensitive CPT2, on the other hand, is located in the mitochondrial matrix and reversibly transfers the acyl chain back to CoA to facilitate beta-oxidation. Although neurons have a relative dearth of CPT1a and CPT1b [2], they express a CPT1 homologue, CPT1c [4].

CPT1c has a high primary amino acid sequence similarity and identity to the canonical CPT enzymes. Therefore, it was surprising that definitive acyltransferase activity or enhanced oxidation of fatty acids could not be shown for CPT1c [4-6]. CPT1c KO mice exhibit both behavioral and metabolic deficits [6-9]. Over-expression of CPT1c in the brain of developing transgenic mice results in microencephaly [10]. Therefore, it is clear that CPT1c plays an important role in brain function. Although there were several metabolites identified that have been altered after over-expression [10,11] or knockout of CPT1c [7], the reaction that CPT1c catalyzes has remained elusive.

Here we used an unbiased metabolomic approach to broadly understand the consequence of CPT1c deletion to gain insight into the biochemical and physiological roles of CPT1c function. Similar to previous work in heterologous systems, we did not see changes consistent with a role for CPT1c in long chain fatty acid beta oxidation. However, there were changes in several fatty acid derived metabolites including endocannabinoids, which may explain the suppressed food intake in these models. Also, some of the most abundant changes were in redox biochemistry consistent with several models of CPT1c function recently proposed.

## Methods

### Animals

Mice with a targeted knockout of exons 1 and 2 of the *cpt1c* gene were propagated and genotyped as previously described [5,6]. Mice were fed a standard lab chow (Harlan 2018) after weaning. All procedures were performed in accordance with the National Institutes of Health Guide for the Care and Use of Laboratory Animals and under the approval of the Johns Hopkins Medical School Animal Care and Use Committee.

### Western blot analysis

A polyclonal rabbit antibody against CPT1c was used as a primary antibody for CPT1c detection in WT and CPT1c KO mice [5,6]. Anti-rabbit horseradish peroxidase (HRP) was used as a secondary antibody, and the blots for CPT1c were developed using ECL reagent. Mouse monoclonal anti-HSC70 (Santa Cruz biotech) and mouse monoclonal anti beta-actin (Sigma) was used as primary antibodies for loading control. Cy3 conjugated fluorescent secondary antibody was used for both HSC70 and beta-actin antibodies.

### Metabolomic measurements and profiling

Unbiased metabolomics analysis of whole brain samples from WT and CPT1c KO mice (n=8/group) that were fasted overnight was performed using liquid chromatography/tandem mass spectrometry (HPLC/MS/MS^2^) and gas chromatography/mass spectrometry (GC/MS) platforms. The platform was able to screen and identify several metabolites in multiple classes, such as amino acids, lipids, and nucleotides. A complete list of the metabolites identified in this study is given in Tables 1, 2, 3 and 4. General platform methods about metabolomic measurements and profiling are described in the metabolomic study done by Eckel-Mahan et al. [12]

**Table 1:** Biochemicals involved in lipid metabolic pathways

**PATHWAY**	**SUB PATHWAY**	**BIOCHEMICAL NAME**	**KEGG**	**CPT1c KOCPT1c WT**	**Welch's Two-Sample*t*-Test**	**CAS**	**PUBCHEM**
**Lipid**	**Essential fatty acid**	linoleate (18:2n6)	C01595	0.93	0.4643	60-33-3;	5280450
		linolenate [alpha or gamma; (18:3n3 or 6)]	C06427	1.04	0.4808		
		dihomo-linolenate (20:3n3 or n6)	C03242	**0.81**	0.0608		5312529
		eicosapentaenoate (EPA; 20:5n3)	C06428	0.72	0.0236	10-2005-9;10417-94-4;	446284
		docosapentaenoate (n3 DPA; 22:5n3)	C16513	**0.80**	0.0662	2234-74-4;	
		docosapentaenoate (n6 DPA; 22:5n6)	C06429	0.77	0.3030	25182-74-5;	6441454
		docosahexaenoate (DHA; 22:6n3)	C06429	0.89	0.2879	6217-54-5;	445580
	**Medium chain fatty acid**	caproate (6:0)	C01585	0.98	0.5408	142-62-1;	8892
		caprylate (8:0)	C06423	0.99	0.9309	124-07-2;	379
		pelargonate (9:0)	C01601	0.89	0.1531	112-05-0;	5461016
		laurate (12:0)	C02679	1.01	0.9051	143-07-7;	3893
	**Long chain fatty acid**	myristate (14:0)	C06424	0.99	0.8942	544-63-8;	11005
		myristoleate (14:1n5)	C08322	1.26	0.1786	544-64-9;	5281119
		palmitate (16:0)	C00249	**0.85**	0.0781	57-10-3;	985
		palmitoleate (16:1n7)	C08362	0.93	0.3794	373-49-9;	445638
		margarate (17:0)		0.86	0.2288	506-12-7;	10465
		10-heptadecenoate (17:1n7)		0.81	0.1051	29743-97-3;	5312435
		stearate (18:0)	C01530	0.94	0.4536	57-11-4;	5281
		oleate (18:1n9)	C00712	0.88	0.2434	112-80-1;	445639
		10-nonadecenoate (19:1n9)		0.72	0.0470	73033-09-7;	5312513
		eicosenoate (20:1n9 or 11)		0.78	0.1453		
		dihomo-linoleate (20:2n6)	C16525	**0.75**	0.0804	2091-39-6;	6439848
		arachidonate (20:4n6)	C00219	0.94	0.4832	506-32-1;	444899
		docosadienoate (22:2n6)	C16533	0.84	0.3185	7370-49-2;	5282807
		adrenate (22:4n6)	C16527	0.81	0.1467	2091-25-0;	5282844
	**Fatty acid, ester**	n-Butyl Oleate		0.96	0.7046	142-77-8;	5354342
	**Fatty acid, dicarboxylate**	2-hydroxyglutarate	C02630	1.00	0.8616	40951-21-1;	43
	**Fatty acid, amide**	oleamide		1.22	0.7962	301-02-0;	5283387
		stearamide	C13846	1.19	0.6546	124-26-5;	31292
	**Eicosanoid**	prostaglandin D2	C00696	1.20	0.1092	41598-07-6;	448457
		prostaglandin E2	C00584	0.93	0.3928	363-24-6;	5280360
		5-HETE		0.99	0.8472	73307-52-5;	9862886
		15-HETE	C04742	0.83	0.9669	54845-95-3;	5280724
	**Endocannabinoid**	palmitoyl ethanolamide		0.64	0.0331		4671
	**Fatty acid & BCAA metabolism**	propionylcarnitine	C03017	1.08	0.4494	17298-37-2;	107738
	**Carnitine metabolism**	carnitine	C00487	0.88	0.0084	461-05-2;	288
		3-dehydrocarnitine*	C02636	1.22	0.0103	10457-99-5;	6991982
		acetylcarnitine	C02571	0.89	0.2172	5080-50-2;	7045767
		oleoylcarnitine		0.83	0.3694		
	**Fatty alcohol, long chain**	1-octadecanol	D01924	1.01	0.8513	112-92-5;	8221
	**Glycerolipid metabolism**	choline phosphate	C00588	0.97	0.4914	72556-74-2;	1014
		ethanolamine	C00189	1.10	0.4703	141-43-5;	
		phosphoethanolamine	C00346	1.06	0.5812	1071-23-4;	52,323,241,015
		glycerol	C00116	0.97	0.6203	56-81-5;	753
		glycerol 3-phosphate (G3P)	C00093	0.98	0.6926	29849-82-9;	754
		glycerophosphorylcholine (GPC)	C00670	0.96	0.9071	28319-77-9;	657272
		cytidine 5'-diphosphocholine	C00307	**1.25**	0.0583	33818-15-4;	13805
	**Inositol metabolism**	myo-inositol	C00137	**0.94**	0.0882	87-89-8;	
		chiro-inositol		0.77	0.1568	643-12-9;	
		inositol 1-phosphate (I1P)		1.01	0.8432	106032-59-1;	
		scyllo-inositol	C06153	0.90	0.1635	488-59-5;	
	**Ketone bodies**	3-hydroxybutyrate (BHBA)	C01089	1.23	0.2197	625-72-9;	441
	**Lysolipid**	1-palmitoylglycerophosphoethanolamine		1.12	0.8591		9547069
		2-palmitoylglycerophosphoethanolamine*		0.83	0.1926		
		1-stearoylglycerophosphoethanolamine		1.20	0.7882	69747-55-3;	9547068
		1-oleoylglycerophosphoethanolamine		1.14	0.8654		9547071
		2-oleoylglycerophosphoethanolamine*		1.07	0.9602		
		1-arachidonoylglycerophosphoethanolamine*		1.06	0.8488		
		2-arachidonoylglycerophosphoethanolamine*		0.45	0.2213		
		2-docosahexaenoylglycerophosphoethanolamine*		0.48	0.3141		
		1-palmitoylglycerophosphocholine		0.47	0.1450	17364-16-8;	86554
		2-palmitoylglycerophosphocholine*		0.59	0.2106		
		1-stearoylglycerophosphocholine		0.51	0.1452	19420-57-6;	497299
		2-stearoylglycerophosphocholine*		1.00			10208382
		1-oleoylglycerophosphocholine		0.56	0.1923	19420-56-5;	16081932
		2-oleoylglycerophosphocholine*		0.65	0.3441		
		1-arachidonoylglycerophosphocholine*	C05208	1.00			
		2-arachidonoylglycerophosphocholine*		0.89	0.4485		
		1-docosahexaenoylglycerophosphocholine*		1.00			
		2-docosahexaenoylglycerophosphocholine*		0.86	0.4614		
		1-palmitoylglycerophosphoinositol*		0.85	0.2160		
		1-stearoylglycerophosphoinositol		0.77	0.1315		
		1-arachidonoylglycerophosphoinositol*		0.87	0.3521		
		1-oleoylglycerophosphoserine		0.92	0.6515		9547099
		2-oleoylglycerophosphoserine*		0.80	0.1921		
		1-palmitoylplasmenylethanolamine*		1.23	0.5225		
	**Monoacylglycerol**	1-palmitoylglycerol (1-monopalmitin)		0.83	0.1685	542-44-9;	14900
		1-stearoylglycerol (1-monostearin)	D01947	0.92	0.3625	123-94-4;	24699
		2-stearoylglycerol (2-monostearin)		0.75	0.1774	621-61-4;	79075
		1-oleoylglycerol (1-monoolein)		0.80	0.1139	111-03-5;	5283468
		2-oleoylglycerol (2-monoolein)		**0.59**	0.0769	3443-84-3;	5319879
	**Sphingolipid**	sphingosine	C00319	0.71	0.3009	123-78-4;	5353955
		palmitoyl sphingomyelin		0.84	0.1297		9939941
		stearoyl sphingomyelin	C00550	1.07	0.2147	85187-10-6;85187-10-6;	6453725
	**Mevalonate metabolism**	3-hydroxy-3-methylglutarate	C03761	1.07	0.4426	503-49-1;	5459993
	**Sterol/Steroid**	cholesterol	C00187	1.00	0.9987	57-88-5;	6432564
		7-alpha-hydroxycholesterol	C03594	1.24	0.2998	566-27-8;	107722
		7-beta-hydroxycholesterol	C03594	1.11	0.2969	566-27-8;	473141
		24(S)-hydroxycholesterol	C13550	0.94	0.5728	2140-46-7;	
		corticosterone	C02140	0.59	0.2402	50-22-6;	5753

**Table 2:** Biochemicals in the amino acid and peptide pathways

**PATHWAY**	**SUB PATHWAY**	**BIOCHEMICAL NAME**	**KEGG**	**CPT1c KOCPT1c WT**	**Welch's Two-Sample*t*-Test**	**CAS**	**PUBCHEM**
**Amino acid**	**Glycine, serine and threonine metabolism**	glycine	C00037	0.91	0.1984	56-40-6;	5,257,127,750
		serine	C00065	0.98	0.6400	56-45-1;	59,516,857,581
		N-acetylserine		1.16	0.2513	97-14-3;	65249
		homoserine	C00263,C02926	1.04	0.5460	672-15-1;	126,476,971,022
		3-phosphoserine	C01005	1.06	0.4516	407-41-0;	
		threonine	C00188	0.98	0.8340	72-19-5;	69,710,196,288
		allo-threonine	C05519	0.98	0.7264	28954-12-3;	992,896,995,276
		betaine	C00719	0.62	0.0393	107-43-7;	247
	**Alanine and aspartate metabolism**	alanine	C00041	0.99	0.8540	56-41-7;	59,507,311,724
		beta-alanine	C00099	0.95	0.7707	56-41-7;107-95-9;	2,394,755,801
		N-acetylalanine	C02847	0.96	0.7172	97-69-8;	88064
		aspartate	C00049	1.02	0.5759	56-84-8;	5960
		N-acetylaspartate (NAA)	C01042	0.98	0.7849	997-55-7;997-55-7;	65065
	**Glutamate metabolism**	glutamate	C00025	1.10	0.1218	56-86-0;	611
		glutamine	C00064	0.96	0.3866	56-85-9;	69,920,865,961
		gamma-aminobutyrate (GABA)	C00334	1.07	0.4581	56-12-2;	6,992,099,119
		N-acetylglutamate	C00624	1.21	0.1108	5817-08-3;	1549099
		N-acetyl-aspartyl-glutamate (NAAG)	C12270	1.04	0.6033	3106-85-2;	5255
		N-acetylglutamine	C02716	0.79	0.1871	2490-97-3;	182230
	**Histidine metabolism**	histidine	C00135	1.11	0.1815	5934-29-2;	7,733,651,426
	**Lysine metabolism**	lysine	C00047	**0.81**	0.0655	56-87-1;	5962
		2-aminoadipate	C00956	0.99	0.9856	542-32-5;1118-90-7;	469
		pipecolate	C00408	0.91	0.4383	4043-87-2;	849
		glutaroyl carnitine		0.77	0.0244	102636-82-8;	
	**Phenylalanine & tyrosine metabolism**	phenylalanine	C00079	**0.93**	0.0731	63-91-2;	69,256,656,140
		tyrosine	C00082	1.10	0.1569	60-18-4;	60,576,942,100
		3-(4-hydroxyphenyl)lactate	C03672	1.28	0.1580	6482-98-0;	9378
	**Tryptophan metabolism**	tryptophan	C00078	1.10	0.1009	73-22-3;	69,235,166,305
		C-glycosyltryptophan*		1.00	0.9578		
		5-hydroxyindoleacetate	C05635	0.99	0.9982	54-16-0;	1826
	**Valine, leucine and isoleucine metabolism**	isoleucine	C00407	0.99	0.7705	73-32-5;	791
		leucine	C00123	0.92	0.1061	61-90-5;	70,457,986,106
		valine	C00183	1.00	0.9896	72-18-4;	69,710,186,287
		alpha-hydroxyisovalerate		0.92	0.9081	600-37-3;	99823
		2-methylbutyroylcarnitine		0.98	0.7985	31023-25-3;	6426901
		isovalerylcarnitine		0.90	0.1479		6426851
		hydroxyisovaleroyl carnitine		**0.90**	0.0807	99159-87-2;	
	**Cysteine, methionine, SAM, taurine metabolism**	cysteine	C00097	**1.13**	0.0835	52-90-4;56-89-3;	58,626,419,722
		cystine	C00491	0.86	0.5529	56-89-3;	595
		taurine	C00245	1.03	0.7783	107-35-7;	11,234,068,592
		S-adenosylhomocysteine (SAH)	C00021	0.96	0.4778	979-92-0;	
		methionine	C00073	0.95	0.1654	63-68-3;	69,920,876,137
		N-acetylmethionine	C02712	0.88	0.1362	65-82-7;	448580
		2-hydroxybutyrate (AHB)	C05984	1.23	0.5077	3347-90-8;	440864
	**Urea cycle; arginine-, proline-, metabolism**	arginine	C00062	**0.95**	0.0964	1119-34-2;	5,246,487,232
		ornithine	C00077	0.90	0.2453	3184-13-2;	6262
		urea	C00086	0.71	0.2913	57-13-6;	117,616,150,869
		proline	C00148	0.97	0.6099	147-85-3;	1,457,426,971,047
		N-acetylornithine	C00437	1.26	0.3497	6205-08-9;	6,992,102,439,232
		trans-4-hydroxyproline	C01157	1.03	0.6431	51-35-4;	58,106,971,053
		argininosuccinate	C03406	0.86	0.3803	156637-58-0;	828
	**Creatine metabolism**	creatine	C00300	1.03	0.2564	57-00-1;	586
		creatinine	C00791	1.20	0.1694	60-27-5;	588
	**Butanoate metabolism**	2-aminobutyrate	C02261	1.03	0.8503	1492-24-6;	4,396,916,971,251
	**Polyamine metabolism**	5-methylthioadenosine (MTA)	C00170	1.08	0.2023	2457-80-9;	439176
		putrescine	C00134	0.83	0.4688	110-60-1;	
		spermidine	C00315	1.04	0.6645	124-20-9;	1102
		spermine	C00750	0.99	0.4470	71-44-3;	1103
	**Guanidino and acetamido metabolism**	4-guanidinobutanoate	C01035	0.98	0.7911	463-003;463-00-3;	500
	**Glutathione metabolism**	glutathione, reduced (GSH)	C00051	1.53	0.1024	70-18-8;	124886
		5-oxoproline	C01879	0.86	0.0291	98-79-3;	7405
		glutathione, oxidized (GSSG)	C00127	2.15	0.0307	103239-24-3;	6,535,911,215,652
		cysteine-glutathione disulfide		**1.33**	0.0802	13081-14-6;	4247235
**Peptide**	**Dipeptide derivative**	carnosine	C00386	0.98	0.8057	305-84-0;	4,392,246,992,100
		homocarnosine	C00884	1.00	0.9807	3650-73-5;	10243361
	**gamma-glutamyl**	gamma-glutamylleucine		0.91	0.1529	2566-39-4;	151023
		gamma-glutamylglutamate		1.24	0.1880	1116-22-9;	92865
		gamma-glutamylglutamine		0.93	0.4450	10148-81-9;	150914
		gamma-glutamylphenylalanine		0.93	0.5544	7432-24-8;	111299

**Table 3:** Biochemicals from the carbohydrate and energy pathways

**PATHWAY**	**SUB PATHWAY**	**BIOCHEMICAL NAME**	**KEGG**	**CPT1c KOCPT1c WT**	**Welch's Two-Sample*t*-Test**	**CAS**	**PUBCHEM**
**Carbohydrate**	**Aminosugars metabolism**	N-acetylglucosamine	C00140	1.03	0.7477	7512-17-6;	24139
		erythronate*		0.98	0.7434	13752-84-6;	2781043
		N-acetylneuraminate	C00270	1.03	0.4494	131-48-6;	
	**Fructose, mannose, galactose, starch, and sucrose metabolism**	fructose	C00095	0.98	0.8393	57-48-7;	5984
		mannose	C00159	0.94	0.6417	3458-28-4;	161658
		mannose-6-phosphate	C00275	0.97	0.7187	70442-25-0;104872-94-8;	
		sorbitol	C00794	0.92	0.5926	6706-59-8;	107428
	**Glycolysis, gluconeogenesis, pyruvate metabolism**	1,5-anhydroglucitol (1,5-AG)	C07326	0.95	0.7426	154-58-5;	
		glycerate	C00258	0.96	0.4928	600-19-1;	752
		glucose-6-phosphate (G6P)	C00668	0.96	0.5074	103192-55-8;	
		glucose	C00293	0.86	0.1984	50-99-7;	79025
		fructose-6-phosphate	C05345	0.83	0.1261	103213-47-4;	
		Isobar: fructose 1,6-diphosphate, glucose 1,6-diphosphate		0.98	0.8050		
		3-phosphoglycerate	C00597	0.80	0.1220	80731-10-8;	
		dihydroxyacetone phosphate (DHAP)	C00111	1.02	0.6910	102783-56-2;	4643300
		1,3-dihydroxyacetone	C00184	1.12	0.4601	62147-49-3;	670
		pyruvate	C00022	0.83	0.0193	127-17-3;	107735
		lactate	C00186	1.06	0.3677	79-33-4;	612
	**Nucleotide sugars, pentose metabolism**	arabitol	C00474	1.30	0.0435	488-82-4;	94154
		ribitol	C00474	0.86	0.1732	488-81-3;	
		sedoheptulose-7-phosphate	C05382	0.91	0.4130	2646-35-7;	616
		ribose 5-phosphate	C00117	1.39	0.0353	18265-46-8;108321-05-7;	447634
		Isobar: ribulose 5-phosphate, xylulose 5-phosphate		1.06	0.5400		
		arabinose	C00181	1.08	0.5432	28697-53-2;	66308
**Energy**	**Krebs cycle**	citrate	C00158	1.02	0.5785	77-92-9;	311
		alpha-ketoglutarate	C00026	0.79	0.2702	305-72-6;328-50-7;22202-68-2;	51
		succinate	C00042	0.88	0.5010	110-15-6;	1110
		fumarate	C00122	0.94	0.5055	100-17-8;	
		malate	C00149	1.11	0.2256	6915-15-7;	525
	**Oxidative phosphorylation**	phosphate	C00009	0.98	0.3284	7664-38-2;	1061
		pyrophosphate (PPi)	C00013	0.84	0.4801	1466-09-3;	644102

**Table 4:** Biochemicals in nucleotide, cofactors and vitamins, and xenobiotic Pathways

**PATHWAY**	**SUB PATHWAY**	**BIOCHEMICAL NAME**	**KEGG**	**CPT1c KOCPT1c WT**	**Welch's Two-Sample*t*-Test**	**CAS**	**PUBCHEM**
**Nucleotide**	**Purine metabolism, (hypo)xanthine/inosine containing**	xanthine	C00385	1.02	0.7727	69-89-6;	1188
		hypoxanthine	C00262	0.98	0.4343	68-94-0;	790
		inosine		1.00	0.8754	58-63-9;	
	**Purine metabolism, adenine containing**	adenine	C00147	**1.11**	0.0801	73-24-5;	190
		adenosine	C00212	0.86	0.1407	58-61-7;	60961
		N1-methyladenosine	C02494	0.94	0.3601	15763-06-1;	5460178
		adenosine 2'-monophosphate (2'-AMP)	C00946	1.00		130-49-4;	
		adenosine 5'-monophosphate (AMP)	C00020	0.89	0.2000	149022-20-8;	15938965
	**Purine metabolism, guanine containing**	guanosine	C00387	1.01	0.9130	118-00-3;	6802
	**Purine metabolism, urate metabolism**	urate	C00366	1.06	0.4983	69-93-2;120K5305;	
		allantoin	C02350	0.76	0.1685	97-59-6;	204
	**Pyrimidine metabolism, cytidine containing**	cytidine	C00475	0.94	0.1562	65-46-3;	6175
		cytidine 5'-monophosphate (5'-CMP)	C00055	1.01	0.8988	63-37-6;	7058165
	**Pyrimidine metabolism, orotate containing**	orotate	C00295	0.86	0.2325	50887-69-9;	967
	**Pyrimidine metabolism, uracil containing**	uracil	C00106	0.97	0.5212	66-22-8;	1174
		uridine	C00299	0.91	0.0141	58-96-8;	6029
		pseudouridine	C02067	0.99	0.7648	1445-07-4;	
	**Purine and pyrimidine metabolism**	methylphosphate		0.85	0.1460	7023-27-0;	13130
**Cofactors and vitamins**	**Ascorbate and aldarate metabolism**	ascorbate (Vitamin C)	C00072	0.87	0.1924	134-03-2;	
		dehydroascorbate	C05422	1.70	0.2338	490-83-5;	835
		threonate	C01620	0.96	0.5529	70753-61-6;	151152
	**Hemoglobin and porphyrin**	heme*	C00032	0.69	0.3695	14875-96-8;	
	**Nicotinate and nicotinamide metabolism**	nicotinamide	C00153	1.00	0.9275	98-92-0;	936
		nicotinamide adenine dinucleotide (NAD+)	C00003	0.87	0.0469	53-84-9;	1,089,765,158,925,280,000
	**Pantothenate and CoA metabolism**	pantothenate	C00864	0.94	0.7951	137-08-6;	6613
		phosphopantetheine	C01134	**0.85**	0.0841	NA;	115254
	**Pyridoxal metabolism**	pyridoxal	C00250	1.05	0.5803	65-22-5;	1050
	**Riboflavin metabolism**	flavin adenine dinucleotide (FAD)	C00016	0.93	0.1085	146-14-5;84366-81-4;	643975
		riboflavin (Vitamin B2)	C00255	0.93	0.2187	83-88-5;	493570
		flavin mononucleotide (FMN)	C00061	0.96	0.7167	130-40-5;	710
	**Tocopherol metabolism**	alpha-tocopherol	C02477	1.04	0.6234	59-02-9;10191-41-0;	14985
**Xenobiotics**	**Chemical**	glycolate (hydroxyacetate)	C00160	1.06	0.7194	79-14-1;	3,698,251,757
		glycerol 2-phosphate	C02979,D01488	1.02	0.9683	819-83-0;	2526
		2-phenoxyethanol		0.94	0.9231	122-99-6;	
		2-pyrrolidinone		0.84	0.6590	616-45-5;	12025
	**Food component/Plant**	ergothioneine	C05570	**0.88**	0.0968	58511-63-0;	3032311
	**Sugar, sugar substitute, starch**	erythritol	C00503	**0.89**	0.0966	149-32-6;	

### Statistical analysis

Pair-wise comparisons between CPT1c WT and KO were performed using Welch’s two-sample t-tests. From the *p*-values, any value below the significance level of 0.05 was interpreted as statistically significant.

## Results

### Carnitine Palmitoyltransferase-1c KO mice

**Figure 1:**
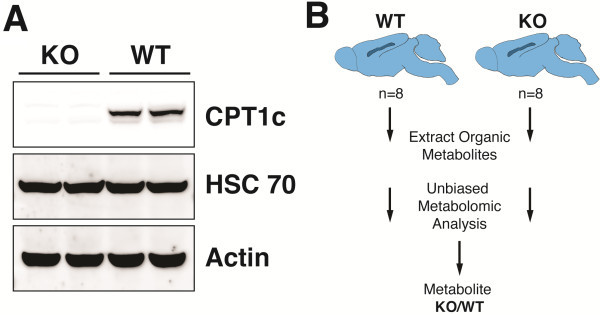
**CPT1c KO mice and metabolomic profiling.** (**A**) CPT1c protein from homogenized brains of WT and CPT1c KO mice were analyzed by western blot using the anti-CPT1c antibody. Hsc70 and Actin were used for loading controls. (**B**) A schematic pathway of metabolomic profiling for KO and WT brains. A commercial supplier of metabolic analysis homogenized 8 brain samples from independent mice to extract organic metabolites for performing unbiased metabolomic analysis using a mixture of GC-MS and LC-MS/MS.

Although CPT1c is widely expressed in transformed cells and tumors [13], we have only been able to reliably detect CPT1c in neurons *in vivo.* To understand the endogenous function of CPT1c, we performed metabolomic profiling on brains of CPT1c KO mice and their littermate controls. Therefore, we collected and snap froze the brains of CPT1c KO and WT littermate sex matched adult mice after an overnight fast. Western blot analysis of WT and CPT1c KO mice showed that KO mice were indeed completely deficient of CPT1c (Figure 1A). These samples were then homogenized and the small organic metabolites were extracted and analyzed by a mixture of GC-MS and LC-MS/MS by a commercial supplier of metabolomic analyses (Figure 1B). Below, we detail the changes in steady-state biochemicals between WT and KO brains that were identified through an unbiased metabolomic screen.

### Fatty acid oxidative metabolites show no difference in overall trend in CPT1c KO mice

**Figure 2:**
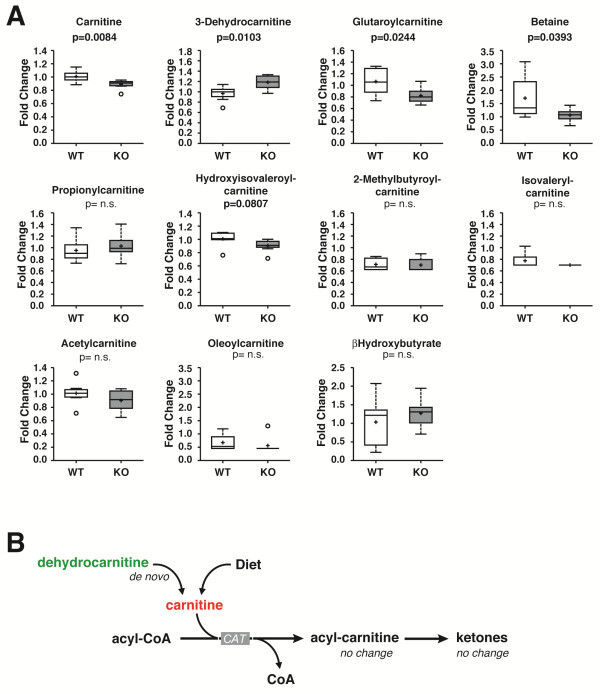
**Loss of CPT1c results in decreased free carnitine and no change in fatty acid oxidative metabolites in the brain.** (**A**) Biochemicals involved in carnitine, amino acid, and fatty acid metabolism from WT and CPT1c KO brains were compared through metabolomic analyses, revealing a statistically significant change in levels of free carnitine (p=0.084), 3-dehydrocarnitine (p=0.0103), glutaroylcarnitine (p=0.0244) and betaine (p=0.0383). (**B**) Schematic of biochemical pathways altered in CPT1c KO mice. Based on this schematic pathway, glutaroyl carnitine and betaine may affect the level of free carnitine, since these biochemicals play a role in carnitine biosynthesis.

Given the high primary amino acid homology of CPT1c to other CPTs, it would follow that CPT1c may be involved in fatty acid beta oxidation or at least in long chain acyl-CoA metabolism. If CPT1c was involved in fatty acid oxidation, we would expect that the deletion of CPT1c would decrease the level of acyl-carnitines and potentially increase the levels of other long chain acyl-CoA dependent biosyntheses. A broad range of lipid species were identified in the metabolomic screen (Table 1). No changes were seen in oleoyl-carnitine, beta-hydroxybutyrate, or acetyl-carnitine, as we would have expected (Figure 2A). However, the metabolomic analysis did show that free carnitine, 3-dehydrocarnitine, glutaroylcarnitine, and betaine were significantly changed (Figure 2A).

Among the metabolites that showed a statistically significant difference, only 3-dehydrocarnitine increased in CPT1c KO mice while glutaroyl carnitine, betaine and free carnitine decreased. Glutaroyl carnitine and betaine are biochemicals that are involved in carnitine biosynthesis (Figure 2B; Table 2). Glutaroyl carnitine is involved in lysine metabolism, which is one of the amino acids that is used to synthesize carnitine. In the carnitine biosynthesis pathway, betaine takes the form of butyrobetaine to synthesize L-carnitine [14]. As a result, it is possible that the decrease in glutaroyl carnitine and betaine could have caused free carnitine levels to decrease in CPT1c KO mice. Previous studies also tested hypothalamic and cortical explants from WT and CPT1c KO mice for their ability to oxidize fatty acids, but there was no evidence that unique properties in neurons existed to allow activation of fatty acid oxidation by CPT1c [5]. CPT1c over-expressed in heterologous cells in vitro also did not show a change in fatty acid oxidation [5]. Therefore, our results remain consistent with previous findings that CPT1c, although it is highly homologous with its isoforms CPT1a and CPT1b, does not participate substantially in neuronal mitochondrial fatty acid oxidation.

### Loss of CPT1c results in decreased levels of endogenous endocannabinoids

**Figure 3:**
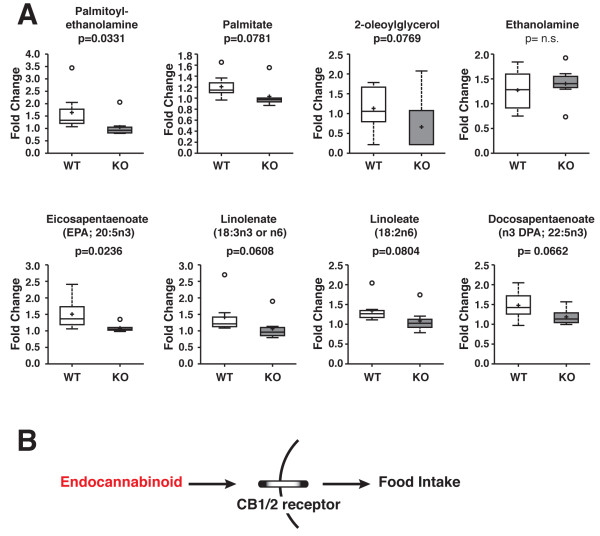
**Loss of CPT1c results in decreased endocannabinoids in the brain.** (**A**) Biochemicals involved in fatty acid biochemistry from WT and CPT1c KO mouse brains were compared to determine if metabolomic analyses showed any statistically significant changes. There was an overall decreasing trend in endocannabinoids in CPT1c KO mice. Specifically, eicosapentaenoate (p=0.0236) and palmitoylethanolamine (p=0.0331) significantly decreased in CPT1c KO mice. (**B**) A schematic of how a decrease in endocannabinoids can induce a decrease in food intake by interacting with CB1 and CB2 cannabinoid receptors.

Several studies have investigated the neurological role of endocannabinoids on food intake [15]. A study investigated the role of endocannabinoids in regulating food intake in the tongue, gut and different brain regions, suggesting that the cannabinoid system plays a role in modulating the activity of neural pathways that regulate food intake and energy expenditure [15]. The brain cannabinoid system, as shown in Figure 3B, regulates food intake through the interaction of endogenous ligands and cannabinoid receptors. From our metabolomic analyses, there was a significant decrease in palmitoylethanolamine and a trend for a decrease in 2-oleolylglycerol in CPT1c KO mouse brains compared to WT mouse brains (Figure 3). There was no significant difference between WT and CPT1c KO mice for free nonesterified fatty acids (Table 1). Among the metabolites shown in Figure 3A, eicosapentaenoate and palmitoylethanolamine showed a significant decrease in CPT1c KO mice with a p-value of 0.0236 and 0.0331, respectively. There was also a slight increase in ethanolamine between WT and CPT1c KO mice, and decrease in 2-oleoylglycerol (p=0.0769), an endogenous cannabinoid (CB) CB-1 agonist (Figure 3A).

### Loss of CPT1c results in increased levels of glutathione

**Figure 4:**
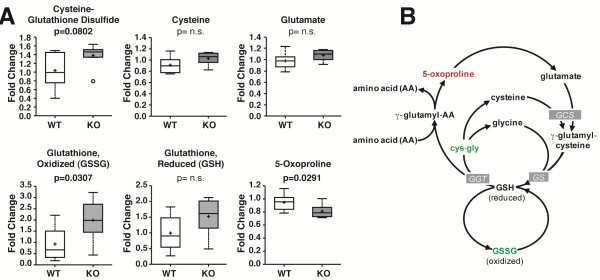
**Loss of CPT1c results in elevated oxidative demands in the brain.** (**A**) In a comparison of biochemicals involved in redox homeostasis in WT and CPT1c KO mouse brains, GSSG and 5-oxoproline were statistically significant. GSSG levels increased in CPT1c KO mice with a p-value of 0.0307, while 5-oxoproline decreased in KO mice (p=0.0291). The biochemicals shown displayed an overall increasing trend in CPT1c KO mice. (**B**) A schematic of the gamma-glutamyl redox cycle. Based on the pathway, an increase in the biochemicals from Figure 4A may cause the cells to become more sensitive to oxidative stress.

The oxidized form of GSH (GSSG) and 5-oxoproline, biochemicals involved in the gamma-glutamyl redox cycle, resulted in a statistically significant difference in CPT1c KO mice (Table 2). GSSG and cysteine-glutathione disulfide levels increased while 5-oxoproline levels decreased in CPT1c KO mice (Figure 4A). Based on the schematic redox pathway shown in Figure 4B, our results suggest that CPT1c may play a role in oxidative metabolism. This is consistent with findings in cancer metabolism. Zaugg et al. depleted the levels of CPT1c in MCF-7 cells to determine whether these cells were sensitive to oxidative stress. Hypoxia was used as a stress inducer, and they found that CPT1c depletion caused an increased sensitivity to oxidative stress, implying that CPT1c may play a crucial role in protecting the cells from stress from the environment [13]. Furthermore, the loss of CPT1c resulted in an increase in ceramides [7,8], a key mediator of oxidative stress [16,17]. However, the mechanism and role of CPT1c in oxidative metabolism remains unknown.

## Discussion

### Role of CPT1c in behavior and physiology

Carnitine acyltransferases are enzymes that catalyze the exchange of acyl groups between carnitine and CoA to facilitate the transport of acyl groups from the cytoplasm to the mitochondrial matrix. Carnitine acetyltransferase (CRAT) and carnitine octonyltransferase (CROT) facilitate transport short- and medium-chain acyl-CoA, while CPT1 facilitate transports long chain acyl-CoA to the mitochondria. CPT1 enzymes are encoded by three genes in mammals that are localized in different tissues and have different properties. CPT1a, which is enriched in the liver, has been heavily studied due to its crucial role in β-oxidation and human fatty oxidation disorders (OMIM #255120) and is lethal when knocked out in mice [18]. CPT1b is localized mainly in the muscle and is a regulator for the use of fatty acids in muscle and is also lethal when knocked out in mice [19]. These two enzymes, which are present on the outer mitochondrial membrane, play a critical role in regulating and facilitating fatty acid beta-oxidation.

The brain specific CPT1c is highly homologous to its closely related genes, CPT1a and CPT1b [4]. However, despite its high homology, CPT1c does not catalyze acyl transfer from long chain acyl-CoA to carnitine [4-6]. Other distinguishing properties of CPT1c include a longer C-terminus and localization in the endoplasmic reticulum (ER) instead of the mitochondria [11]. Although it does not facilitate acyl transfer in the cell, CPT1c most likely remains sensitive to the endogenous allosteric CPT1 inhibitor, malonyl-CoA, binding with a similar affinity as CPT1a [4,6]. Moreover, while other isoenzymes are expressed in a broad range of organisms, CPT1c seems to have risen late in evolution, raising the question whether CPT1c has a specific role in mammalian brain function.

Several studies used CPT1c knockout (KO) and CPT1c transgenic mice to investigate the role of CPT1c in the CNS. Knockout studies showed that loss of CPT1c did not affect the viability or fertility of the mice, but resulted in a suppression in food intake and decrease in body weight when they were fed a normal or low-fat diet [6,9]. Paradoxically, when high fat diet was given to CPT1c KO mice, they exhibited diet-induced obesity which ultimately resulted in a diabetic phenotype [5,6]. Even though fatty acid oxidative metabolites showed no significant change based on the metabolomic analysis, due to a decrease in peripheral energy expenditure CPT1c KO mice were more susceptible to obesity and diabetes when fed a high fat diet. This suggests that CPT1c has a hypothalamic function in protecting the body from adverse weight gain when the mice were fed a high fat diet. Transgenic CPT1c mice (CPT1c-TgN), on the other hand, which allowed conditional expression of CPT1c in a tissue-specific manner via cre-lox recombination, showed enhanced expression of CPT1c and they were protected from diet-induced obesity even on a high-fat diet [10].

CPT1c KO mice also showed impaired spatial learning [7]. Cpt1c deficiency was shown to alter dendritic spine morphology by increasing immature filopodia and reducing mature mushroom and stubby spines. Compared to WT mice, CPT1c KO mice showed a higher escape latency, implying that they had a delay in the acquisition phase [7]. Based on this study, CPT1c deficiency interfered with consolidating new information but did not affect retaining information or motor behavior. As a result, there may be other physiological roles of CPT1c in addition to regulating food intake and energy expenditure consistent with its broad expression throughout the nervous system [7].

### Endocannabinoid regulation of food intake

Endocannabinoids are endogenous ligands that bind to cannabinoid receptors to regulate many aspects of physiology and behavior. Specifically, the brain endocannabinoid system regulates food intake via the hypothalamus, where it activates necessary mediators to induce appetite after a short-term food deprivation. CB1 receptor KO mice showed reduced food intake, similar to CPT1c KO mice [20,21]. Based on our results, CPT1c could be interacting with the cannabinoid system, causing an overall decreasing trend in endocannabinoids in CPT1c KO mice. In this context, the loss of CPT1c could have influenced the endocannabinoid system and its function to regulate food intake and body weight, which may explain the suppressed food intake in CPT1c KO mice [5,9]. Therefore, a decrease in endocannabinoids based on metabolomic profiling may suggest a putative role of the endocannabinoid system in suppressing food intake in CPT1c KO mice. However, it is unclear if CPT1c affects endocannabinoid metabolism directly or more likely indirectly by altering neuronal specific fatty acid metabolism.

### Glutathione and redox metabolism

Neurons are particularly sensitive to oxidative stress and damage caused by reactive oxygen species (ROS). On the cellular level, there are many endogenous metabolic stress inducers, such as ROS produced from the mitochondria and cytosolic enzymes, such as cyclooxygenase and lipoxygenase. There are also various exogenous conditions that can also promote the level of ROS species to increase, such as H_2_O_2_ and hypoxia, that induces irreversible cellular damage or cell death. As shown by the pathway in Figure 4B, reduced glutathione (GSH) and oxidized glutathione (GSSG) are tightly regulated in order to maintain cellular redox homeostasis and to protect the cells from oxidative damage [17]. Carrasco et al. showed that CPT1c expression correlated with ceramide production and loss of CPT1c resulted in reduced ceramide levels. [7]. A recent study on the role of CPT1c in cancer cells in response to metabolic stress showed that CPT1c could participate in protecting cells from stress. In addition, they postulated that metabolic stress could alter regulation of the CPT1c gene, reducing ATP production and increasing sensitivity towards metabolic stress [13]. Here, we showed that CPT1c deficiency results in an increased oxidative environment. This may indicate that although CPT1c does not contribute in large part to beta-oxidation, it may be involved in other neuron specific oxidative metabolism. Alternatively, CPT1c may need to be activated in a yet to identified stress-induced manner. Barger et al. [22] showed that CPT1c was required for leukemia growth under low glucose conditions. Therefore, CPT1c may have a context dependent role in fatty acid catabolism. Although here we show that CPT1c could play a role in oxidative stress, the precise role of CPT1c in relation to oxidative stress remains unknown.

## Conclusion

Unbiased metabolomic profiling of steady-state metabolites in WT and CPT1c KO brains revealed subtle changes in a broad range of metabolites *in vivo*. The metabolic alterations are not consistent with CPT1c playing a role in beta-oxidation or a large non-redundant role in bioenergetics.

## Abbreviations

WT: Wild-type; KO: Knockout; CPT1: Carnitine Palmitoyltransferase 1; CPT2: Carnitine Palmitoyltransferase 2; CoA: Coenzyme A; CB: Cannabinoids; GC: Gas chromatography; MS: Mass spectrometry.

## Competing interests

The authors declare that they have no competing interests.

## Authors’ contributions

MJW conceived of the project, collected samples and aided in writing. JL interpreted results and wrote the manuscript. All authors read and approved the final manuscript.
